# The holistic way of tackling the COVID-19 pandemic: the one health approach

**DOI:** 10.1186/s41182-020-00257-0

**Published:** 2020-08-14

**Authors:** Vivian Mushi

**Affiliations:** Department of Parasitology and Medical Entomology, School of Public Health and Social Sciences, Muhimbili University of Health and Allied Sciences, P.O. Box 65001, Dar es Salaam, Tanzania

**Keywords:** COVID-19, SARS-CoV-2, One health approach, Adoption and benefits

## Abstract

The severe acute respiratory syndrome coronavirus 2 (SARS-CoV-2) that is causing a global pandemic had a zoonotic origin in China. Considering the inter-connectedness between human, environment, and animal health, the One Health approach is the appropriate strategy to control and mitigate the effects of the ongoing coronavirus disease 2019 (COVID-19). This letter explains the benefits of the One Health approach and recommends specific measures that could be taken to accelerate the fight against COVID-19 and prevent the spread of newly emerging infectious diseases.

To the Editor

The novel severe acute respiratory syndrome coronavirus 2 (SARS-CoV-2) is the etiological agent responsible for a respiratory illness known as coronavirus disease 2019 (COVID-19). The World Health Organization (WHO) declared COVID-19 a public health emergency of international concern on 30 January 2020 and, because of the worldwide spread, a pandemic on 11 March 2020 [1]. Evidence to date from virus genome sequencing and evolutionary analysis suggests that the SARS-CoV-2 originated from bat populations, and thus, transmission to humans was possible either directly from bats to humans or indirectly through an unknown intermediate host [2, 3].

Because SARS-CoV-2 has a zoonotic origin, adoption of a One Health approach could be the best mechanism to combat COVID-19. The One Health approach involves multi-sectoral and trans-disciplinary collaborative efforts that work from local, regional, national to global levels to achieve optimal health and well-being of people, animals, and plants in shared environments. Therefore, a One Health approach aims to lower risk and mitigate effects of health crises occurring at the interface between humans, animals, and their environments [4]. The One Health approach is not a new concept. The history goes back to the 1800s, when scientists observed the link between human and animal health especially similarities in disease processes among animals and humans, and the concept was further advanced in the twentieth century due to the threat of emerging pandemics such as the severe acute respiratory syndrome (SARS) and avian influenza (H5N1) [5].

The global community can adopt a One Health approach in the fight against COVID-19 by the following steps. First, conduct an interdisciplinary coordinated surveillance and monitoring during the pandemic with a view to studying the disease patterns and trends, intensity of transmission, geographical spread, and impact of the pandemic for modification of the existing response strategies. Second, establish integrated human and animal health laboratories that will help to strengthen the capacity to conduct integrated studies on COVID-19. Third, collaborate and share protocols, ideas, and guidelines on COVID-19 diagnosis, interpretation of results, a reporting system, and data sharing. This will ensure that the same standards are observed worldwide for diagnosis, reporting, and results sharing. Fourth, governments should coordinate multi-sectoral engagement and partnership with One Health stakeholders in the fight against COVID-19 so as to speed up prevention and disease response [4, 6, 7].

A One Health approach will be beneficial as we fight against COVID-19 in financially constrained settings as it allows cost-sharing in an interdisciplinary field within the responsible ministries. Human and animal health sectors working separately are more costly than when the sectors work collaboratively and share resources [8]. One Health laboratories are crucial for conducting research in order to collect and share data and advance knowledge that will help to reduce ambiguity in COVID-19 mitigation decisions [9]. The One Health approach, through the collaboration of multiple sectors, will help in controlling the disease and will lessen the social and economic impact of the COVID-19 pandemic [10]. The lessons learned from utilizing the One Health approach in the pandemic will be useful for risk reduction and control of emerging and re-emerging infectious diseases.

The current COVID-19 pandemic is an opportune time to emphasize the One Health approach in global health. I wish to recommend the following measures to accelerate the fight against COVID-19. These are to:

i. Mobilize resources that will assist in the implementation of the One Health activities such as surveillance and monitoring of the COVID-19 pandemic; and
ii. Involve communities in the fight against COVID-19. Implementation of the One Health approach without the active involvement of communities will not succeed because an understanding of community knowledge, practices, and customs are fundamental for COVID-19 prevention and control interventions targeting those communities.

In conclusion, although COVID-19 has already spread in many parts of the world, implementing the One Health approach could be the solution to the control of COVID-19. Now more than ever, it is time to adopt, invest in, and embrace the One Health approach because it can help with both the current COVID-19 pandemic and also mitigate the effects of other emerging infectious diseases.

## Data Availability

All the data used are from the references provided.

## Abbreviations

COVID-19: Coronavirus disease 2019
SARS-CoV-2: Severe acute respiratory syndrome coronavirus 2
WHO: World Health Organization
